# Quality of life in early-onset colorectal cancer patients: systematic review

**DOI:** 10.1093/bjsopen/zrad030

**Published:** 2023-05-08

**Authors:** Oliver Waddell, Jared Mclauchlan, Andrew McCombie, Tamara Glyn, Frank Frizelle

**Affiliations:** Department of Surgery, University of Otago Christchurch, Christchurch, New Zealand; Department of Surgery, Te Whatu Ora Health New Zealand Waitaha Canterbury, Christchurch, New Zealand; Department of Surgery, University of Otago Christchurch, Christchurch, New Zealand; Department of Surgery, Te Whatu Ora Health New Zealand Waitaha Canterbury, Christchurch, New Zealand; Department of Surgery, University of Otago Christchurch, Christchurch, New Zealand; Department of Surgery, Te Whatu Ora Health New Zealand Waitaha Canterbury, Christchurch, New Zealand; Department of Surgery, University of Otago Christchurch, Christchurch, New Zealand; Department of Surgery, Te Whatu Ora Health New Zealand Waitaha Canterbury, Christchurch, New Zealand

## Abstract

**Background:**

There is an increasing incidence of early-onset colorectal cancer; however, the psychosocial impacts of this disease on younger adults have been seldom explored.

**Methods:**

A systematic review was conducted according to the PRISMA guidelines. The Cochrane Central Register of Controlled Trials, MEDLINE, Embase, CINAHL, PubMed, and Scopus were searched, and papers were included if published in English within the last 10 years and if they reported results separately by age (including early-onset colorectal cancer, defined as colorectal cancer diagnosed before the age of 50 years). Critical appraisal of all studies was done using the Joanna Briggs Institute tools. The primary outcome of interest was the global quality of life in patients with early-onset colorectal cancer. Secondary outcomes included the effect on sexual function, body image, finances, career, emotional distress, and social and family functioning.

**Results:**

The search yielded 168 manuscripts and 15 papers were included in the review after screening. All studies were observational, and included a total of 18 146 patients, of which 5015 were patients with early-onset colorectal cancer. The studies included scored highly using Joanna Briggs Institute critical appraisal tools, indicating good quality and a low risk of bias, but data synthesis was not performed due to the wide range of scoring systems that were used across the studies. Six papers reported significant negative impacts on quality of life in patients with early-onset colorectal cancer. Three of the four studies that compared the quality of life in patients with early-onset colorectal cancer with older patients found that the younger group had worse mean quality-of-life scores (*P* ≤ 0.05). Secondary outcomes measured in five studies in relation to sexual dysfunction, body image, financial and career impacts, and social and family impacts and in eight studies in relation to emotional distress were found to be more severely impacted in those with early-onset colorectal cancer compared with those with late-onset colorectal cancer.

**Conclusion:**

Whilst data are limited, the impact of colorectal cancer is different in patients with early-onset colorectal cancer compared with older patients in relation to several aspects of the quality of life. This is particularly prominent in areas of global quality of life, sexual functioning, family concerns, and financial impacts.

## Teaser

There is an increasing incidence of early-onset colorectal cancer. Little has been done to explore the distinct psychosocial impacts of this disease on younger adults. The aim of this systematic review was to look at the different psychosocial effects in early-onset colorectal cancer, focusing on the impact on quality of life, sexual function, body image, finances, career, emotional distress, and social and family functioning.

## Introduction

Despite an overall reduction in the incidence of colorectal cancer (CRC), over the past few decades, there has been a marked increase in early-onset colorectal cancer (EOCRC) in many countries in the developed world, for example the USA, New Zealand, Canada, Australia, Scotland, Sweden, and France^1–3^. This increase in EOCRC rates, defined as CRC diagnosed before the age of 50 years in these western populations, was largely driven by the increased incidence of distal colon and rectal carcinoma. In New Zealand, among patients aged under 50 years, the incidence of distal colonic cancer in men increased by 14 per cent per decade, while the incidence of rectal cancer in men increased by 18 per cent and in women by 13 per cent^1,2^. Within the next decade, it is estimated that one in four rectal cancers will be diagnosed in adults younger than 50 years^4^.

A diagnosis of CRC negatively impacts a patient’s quality of life (QOL)^5^. The diagnosis and treatment of CRC have been shown to have ongoing psychological, physical, social, and functional impacts, which can lead to a reduced QOL^6^.

The pathway through cancer diagnosis, treatment, and survivorship is different for younger adults who are at a different stage in their lives, which is likely to affect their QOL in different ways. There are several concerns over how their diagnosis may impact areas such as fertility, sexual function, body image, career aspirations, financial security, and the future impact on their young children^7,8^.

Risk factors for patients with CRC of having a poorer QOL and greater psychological distress include later-stage disease, having a stoma, fatigue, being single, low social support, lower income, and smoking^9,10^. However, these data are derived from research across all ages and not specific to patients with EOCRC and, to date, research into the specific psychosocial effects of EOCRC is limited. Given the increasing incidence of younger patients, the aim of this systematic review was to investigate the QOL spectrum in this subgroup.

The primary aim was to systematically review the available evidence on the psychosocial impact of EOCRC. The primary outcome of interest was global QOL, whereas secondary outcomes included sexual dysfunction, intimate relationships, body image, financial situation, career, emotional distress, and social and family functioning.

## Methods

The systematic review was conducted in accordance with the PRISMA checklist^11^.

### Search strategy and study selection

A literature search of the Cochrane Central Register of Controlled Trials, MEDLINE, Embase, CINAHL, PubMed, and Scopus was performed. Reference lists of identified studies were also searched. Search terms included (colorectal neoplasm* OR colorectal cancer* OR bowel cancer* OR bowel neoplasm*) AND quality of life AND survivor (see the *Supplementary material*).

Original studies reporting the impact of a diagnosis or treatment of colon or rectal cancer on QOL or secondary outcomes in patients aged 18–50 years, between 2012 and January 2023, were included. Further inclusion criteria included publication in English and publication in a peer-reviewed journal.

If studies included patients of all ages, the data had to be reported separately by age, with an upper limit for the age comparison group of 50 years. Any study that reported QOL, either measured by a validated tool or via their own methods, at any time point after diagnosis of cancer, was included.

### Outcomes of interest

The primary outcome was the impact on QOL in EOCRC, defined as CRC diagnosed before the age of 50 years. Secondary outcomes included impacts on sexual dysfunction, intimate relationships, body image, financial situation, career, emotional distress, and social and family functioning. Comparison with late-onset CRC (LOCRC), defined as CRC diagnosed after 50 years, was included.

### Data synthesis and statistics

Given that the outcomes were qualitative, and due to the wide range of scoring systems that were used, quantitative synthesis was not performed to combine results across studies. The results were tabulated and the outcomes summarized. Each study was evaluated according to its design, number and geography of patients, and QOL scoring tool used. Critical appraisal of each study was done using tools provided by the Joanna Briggs Institute (JBI). These tools are designed to help assess the quality and applicability of studies by systematically assessing methodology, analysis, and potential for bias^12^.

## Results

### Search results

**Fig. 1:**
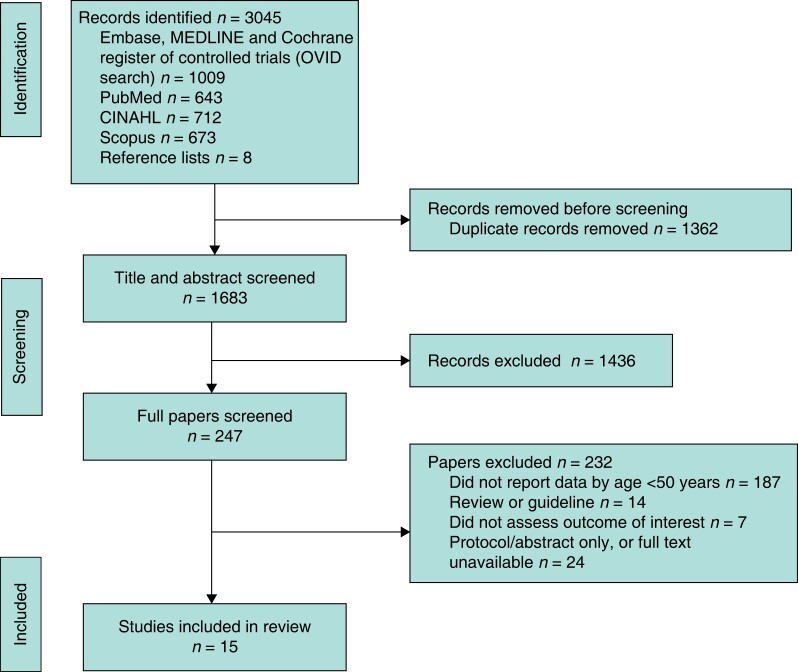
PRISMA diagram

The search yielded a total of 1683 citations after removal of duplications. These titles and abstracts were screened and a total of 247 full papers were reviewed; however, 15 studies were included in the final analysis (*Fig. 1*).

### Study characteristics

**Fig. 2:**
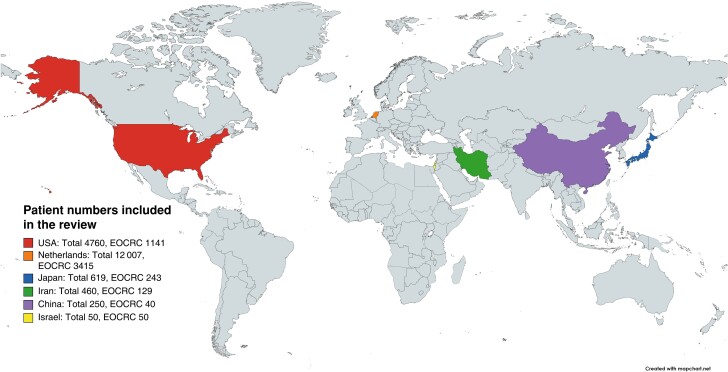
Patient numbers included in the review The location and number of patients with total colorectal cancer and patients with early-onset colorectal cancer (EOCRC) from the studies included in this systematic review. Not included are 1428 patients with EOCRC recruited from the REACCT multinational database.

The included studies were all observational. There were 11 cross-sectional studies^13–23^, two case–control studies^24,25^, one cohort study^26^, and one qualitative study^8^. Of a total of 18 146 patients, 5015 were EOCRC patients. The largest study included was a case–control investigation from the Netherlands including 12 007 patients^25^. The smallest study had 14 participants taking part in in-depth interviews as part of a qualitative study^8^. Four studies included EOCRC patients only, with two of these including only patients under the age of 40 years^8,15,19,21^. The remaining studies included both EOCRC and LOCRC patients, but with varying cut-off ages for their age comparison analysis, with some under 40^14,24^, under 45^25^, or under 50 years^13,16,18,20,23,26^. Studies were mainly from English-speaking countries with eight from the USA^8,13,14,19,20,22,24,26^. Other studies from predominantly non-English-speaking countries were from Israel^15^, the Netherlands^25^, Iran^16,17^, Japan^18^, and China^23^, and one was a multinational collaboration^21^ (*Fig. 2*).

The time from diagnosis to assessment of outcome ranged from at the time of diagnosis through to long-term survivors of greater than 5 years (*Table 1*).

**Table 1:** Study characteristics

Authors	Year	Total number of patients	EOCRC number (% of total)	Time since diagnosis of QOL investigation	Country	Cancer type	Study design and aim
Bailey *et al.*^4^	2014	830	282 (34.0)	>5 years	USA	CRC	Cross-sectional study with a comparison group. Survey of long-term (>5 years) younger-onset CRC survivors, matched 1 : 2 with older controls. Aim to assess differences in functional deficits and symptoms between these two groups.
Sanford *et al.*^24^	2014	718	37 (5.2)	During treatment	USA	CRC and breast cancer. Analysed separately	Case–control study comparing group of young adults with CRC and breast cancer with a control group of older patients. Interviewed at diagnosis with follow up 4–5 weeks later. Aim to assess differences in symptom burden and functional impacts between younger and older patients.
Mack *et al.*^14^	2016	592	148 (25.0)	4–6 months	USA	CRC (80%) and lung cancer	Cross-sectional study with a comparison group. Patients 21–40 years were compared 1 : 3 with patients aged 41–60 years. Aim to establish differences in decision-making preferences, family involvement, and treatment worries.
Perl *et al.*^15^	2016	50	50 (100)	6–24 months	Israel	Gastrointestinal tract cancer, CRC (80%)	Cross-sectional study. Patients <40 years with gastrointestinal tract cancer underwent a phone interview. Aim to evaluate symptoms, general functioning, sexual functioning, and unmet needs in CRC patients. They also included comparison with data from other studies in breast cancer and prostate cancer patients.
Aminisani *et al.*^16^	2017	157	44 (28.0)	0–6 years	Iran	CRC	Cross-sectional study. CRC patients identified from a cancer registry were interviewed. Aim to assess levels of depression and anxiety and their relation to HRQOL.
Adams *et al.*^26^	2016	1021	228 (22.3)	>5 years	USA	CRC	Mixed-methods study. Cross-sectional survey was done to assess HRQOL in CRC patients from a registry. Physical and mental component scores were calculated. This cohort of patients was then followed up to see if those with low (<10%) QOL scores had different rate. Aim to measure HRQOL in CRC survivors and see if this was related to rate.
Blum-Barnett *et al.*^8^	2019	14	14 (100)	Not reported	USA	CRC	Qualitative study. Focused interviews of small group of CRC survivors, all aged <50 years. Aim to assess financial burden and QOL impacts of CRC diagnosis on patients <50 years.
Aminisani *et al.*	2021	303	85 (28.1)	At time of diagnosis	Iran	CRC	Cross-sectional study. Interview of patients admitted to hospital with CRC measuring anxiety and depression. Aim to assess levels of anxiety and depression in CRC patients and assess differences between sexes.
Kobayashi *et al.*^18^	2020	619	243 (39.3)	<6 months–2 years	Japan	CRC (47.5%), stomach cancer, and lung cancer	Cross-sectional study. Questionnaire study completed by adult patients with CRC, stomach cancer, or lung cancer diagnosed. Aim to measure association between health literacy and social support and QOL in cancer patients.
de Wind *et al.*^25^	2021	12 007	3415 (28.4)	2–4 years	Netherlands	CRC	Case–control study. Group of CRC cases from cancer registry matched with population controls, aged 16–62 years at diagnosis. Comparing employment data taken from national tax system. Aim to assess whether CRC survivors have a higher risk of unemployment compared with the general population at diagnosis and up to 4 years later, and which patients are at highest risk of unemployment.
Miller *et al.*^19^	2021	196	196 (100)	6–36 months	USA	CRC	Cross-sectional study. Online survey of CRC patients aged 18–39 years. Secondary analysis to compare those 6–18 months post-diagnosis with those 19–36 months post-diagnosis. Aim to assess QOL in EOCRC patients and its correlation with time since diagnosis.
Boehmer *et al.*^20^	2021	480	71 (14.8)	1–5 years	USA	CRC	Cross-sectional study with a comparison group. Phone interview of CRC survivors in regions that had higher rates of same-sex relationship households, comparing CRC survivors of diverse sexual orientations with heterosexual patients. Aim to assess QOL in CRC and compare results between sexual minority groups and heterosexual patients.
REACCT Collaborative^21^	2022	1428	1428 (100)	Median 42 months	Multinational	Rectal	Cross-sectional study. Patients <50 years diagnosed with a rectal cancer were included, taken from the REACCT Collaborative database measuring functional outcomes. Aim to assess functional outcomes in early-onset rectal cancer patients.
Potosky *et al.*^22^	2022	909	165 (18.2)	Median 9 months	USA	CRC	Cross-sectional study. CRC survivors surveyed to measure eight QOL-related domains. Aim to assess the prevalence and predictors of different QOL-related domains.
Mo *et al.*^23^	2023	250	40 (16.0)	During or immediately after treatment	China	CRC	Cross-sectional study. Chinese CRC patients interviewed while in hospital. Aim to establish the prevalence and influencing factors of financial toxicity in patients with non-metastatic CRC.

EOCRC, early-onset colorectal cancer; QOL, quality of life; CRC, colorectal cancer; HRQOL, health-related quality of life.

### Quality of the studies

The studies were all assessed for quality using the JBI scoring checklists for each relevant study design. Studies were scored on how many of the criteria each study had satisfied after excluding items that were not applicable. All studies satisfied a high proportion of criteria ranging from 87.5 to 100 per cent, implying a high level of quality and a low risk of bias. Results of these assessments can be seen in *Tables S1–S5*.

### Global quality of life

Seven studies reported the impact of CRC diagnosis on global QOL^8,15,16,18–20,22^. A variety of validated scoring systems were used to measure QOL^27,28^. These included short form-12 (SF-12), the Cancer Rehabilitation Evaluation System (CARES), the 30-item European Organisation for Research and Treatment of Cancer Quality of Life Questionaire (EORTC QLQ-C30), the Patient-Reported Outcomes Measurement Information System (PROMIS), Functional Assessment of Cancer Therapy—Colorectal (FACT-C), and Functional Assessment of Cancer Therapy—General (FACT-G). One study used qualitative patient interviews^8^ (*Table 2*).

**Table 2:** Effect on quality of life

Authors	Age of participants (years)	QOL scoring tool used	Key findings relevant to QOL
Perl *et al.* (2016)^15^	All participants <40	SF-12, CARES	Following treatment EOCRC patients experience symptoms and stressors that significantly worsen their QOL and interfere with daily activities including diarrhoea, sleeping disorder, sexual dysfunction, impairment in occupational activities, and difficulty coping with children.
Aminisani *et al.* (2017)^16^	Patients <50 compared with >50	HADS-A, HADS-D, EORTC QLQ-C30	No statistically significant differences in rates of depression, anxiety, or global QOL.
Blum-Barnett *et al.* (2019)^8^	All participants <50	Qualitative patient interviews	EOCRC has significant negative effects on QOL and career due to various emotional and physical side effects. Prominent themes included strained relationships, anxiety, worry about children, lack of information, sexual dysfunction, impacts on financial situation, career trajectory, lost earning potential, and reduced performance at work.
Kobayashi *et al.* (2020)^18^	Patients <50 compared with >50	FACT-G	QOL was lower overall in patients <50 compared with >50 (*P* ≤ 0.001). In older patients higher levels of social support were the strongest predictor of higher QOL, with higher health literacy also having a positive influence. In the group <50 they found no statistically significant association with health literacy or social support and QOL.
Boehmer *et al.* (2021)^20^	Patients under <50 compared with >65	EORTC QLQ-CR29, SF-12	EOCRC patients have statistically significant lower physical QOL (bivariate association of −3.6, *P* ≤ 0.05), and lower, but not statistically significant, mental QOL (bivariate association of −1.11, *P* ≥ 0.05)
Miller *et al.* (2021)^19^	All participants <50	FACT-C	Overall low HRQOL in all EOCRC patients. Emotional and physical well-being was higher in patients who were 19–36 months past diagnosis compared with patients who were 9–18 months past diagnosis, implying some improvement over time. FACT-C QOL scores are significantly lower when compared with data from other studies in older CRC patients (67.7/136 compared with 111.9/136).
Potosky *et al.* (2022)^22^	Patients <40 compared with older patients in 5-year groups	PROMIS	Older patients were found to be progressively less likely to score in the low HRQOL group compared with younger patients (OR = 0.83 (95% c.i. 0.72,0.95), for every 5-year increase in age).

QOL, quality of life; SF-12, short form-12; CARES, Cancer Rehabilitation Evaluation System; EOCRC, early-onset colorectal cancer; HADS, Hospital Anxiety and Depression Scale; EORTC QLQ-C30, 30-item European Organisation for Research and Treatment of Cancer Quality of Life Questionaire; FACT-G, Functional Assessment of Cancer Therapy—General; EORTC QLQ-CR29, 29-item European Organisation for Research and Treatment of Cancer Quality of Life Questionaire SF-12, short form-12; FACT-C, Functional Assessment of Cancer Therapy—Colorectal; HRQOL, health-related quality of life; PROMIS, Patient-Reported Outcomes Measurement Information System.

Three studies involved only younger patients and described the negative impacts on QOL specific to EOCRC^8,15,19^. Four compared global QOL of younger *versus* older patients, with three out of four finding that QOL was more adversely impacted in EOCRC patients^18,20^ and one found no statistically significant difference^16^.

In particular, one study^19^, involving only EOCRC patients aged 18–40 years (between 6 and 36 months after diagnosis), reported that global QOL scores, measured using FACT-C, were a mean(s.d.) of 67.7(13.15), out of a possible 136 (a lower score corresponds to a worse QOL)^27^. Miller *et al*.^19^ also reported that the physical and emotional well-being scores of FACT-C were better in the patients who were between 19 and 36 months after diagnosis compared with between 9 and 18 months, implying that QOL deficits may improve over time, but not to baseline (physical well-being score 16.56 *versus* 14.31 (*P* = 0.001) and emotional well-being score 12.56 *versus* 11.13 (*P* = 0.007) respectively).

Blum-Barnett *et al*.^8^ performed a qualitative study that involved in-depth interviews with EOCRC patients. This study highlighted the distinct effects that this diagnosis had on younger patients, broadly defining impacts as financial impacts (discussed elsewhere in this paper) and QOL impacts. They concluded that there was a significantly negative impact on overall QOL due to both emotional and physical effects.

Another study focused on patients younger than 40 years, concluding that young patients after treatment for gastrointestinal cancers experienced a reduction in QOL due to physical symptoms, particularly diarrhoea, sleep issues, and sexual dysfunction, and, likewise, a reduction in QOL due to functional issues, particularly regarding occupational activities and coping with children^15^.

Other studies included both EOCRC patients and older patients and provided a comparison between the two. Three of the four studies found a more pronounced negative impact on QOL in younger patients, with the remaining study finding no difference. The first one^20^ focused on the different effects of QOL on homosexual patients *versus* heterosexual patients and found that global QOL was worse when comparing patients under 50 years with those over 65 years. Particularly, physical QOL had a significant bivariate association of −3.60 (*P* ≤ 0.05), implying a significantly decreased physical QOL in the younger patients. The mental QOL score was also lower in those under 50 years, but this result did not reach significance^20^.

In another study from Japan^18^, on the relationship between health literacy and social support and QOL in patients with cancer, the authors reported a significantly lower QOL score in patients under 50 years compared with older patients, using FACT-G scores. They also found that QOL in older patients improved with increasing social support and health literacy, but this was not the case with the younger population. An American study also found that patients were progressively less likely to fall into the low health-related QOL group compared with the high health-related QOL group as patients got older (OR = 0.83 (95 per cent c.i. 0.72 to 0.95), per 5-year increase in age)^22^.

There was just one study^16^ that reported no difference. They investigated the relationship between rates of depression and anxiety and health-related QOL. When looking at the total QOL scores using QLQ-C30, there was no statistically significant difference in scores between patients over 50 years and those under 50 years. There were also no differences in the rate of depression or anxiety. They did, however, find that patients with depression and anxiety were more likely to have lower QOL scores^16^.

### Sexual dysfunction, intimate relationships, and body image

Five studies reported on these findings, using a variety of validated scoring tools including European Organisation for Research and Treatment of Cancer (EORTC) QLQ-CR29, MD Anderson Symptom Inventory (MDASI), SF-12, and CARES, and, in the case of one study, using qualitative patient interviews^8,13,15,21,24^ (*Table 3*).

**Table 3:** Impact on sexual dysfunction, intimate relationships, and body image

Author	Age of participants (years)	Scoring tools used	Key findings relevant to sexual dysfunction, intimate relationships, and body image
Bailey *et al.* (2014)^13^	Patients <50 compared with >50	EORTC QLQ-CR29	Younger patients reported worse body image (*P* ≤ 0.05). Older patients experience worse sexual dysfunction and impotence; however, impotence rates were still high in younger patients (63% *versus* 42% (*P* ≤ 0.001)).
Sanford *et al.* (2014)^24^	Participants <40 compared with >40	MDASI	Of younger patients, 24% experienced a moderate to severe impact on their intimate relationships compared with 11% of those >40 (*P* ≤ 0.05).
Perl *et al.* (2016)^15^	All participants <40	SF-12, CARES	Of patients, 40% reported an impact on sexual functioning, which improved with time, but never to baseline levels. Sexual functioning scores in men, when compared with data on prostate cancer patients, are in the bottom 41%. Compared with the normal population they score in the bottom 17%. In women, sexual dysfunction when compared with breast cancer patients was equivalent to the bottom 13% worst affected.
Blum-Barnett *et al.* (2019)^8^	All participants <50	Qualitative patient interviews	Prominent themes included the negative impact cancer diagnosis and treatment had on intimate relationships and sexual dysfunction. Patients wanted more communication around sexual dysfunction.
REACCT Collaborative (2022)^21^	All participants <50	Not reported	The second most common functional issue reported was sexual dysfunction (6%). Very small numbers (*n* = 15/1428) reported fertility issues.

EORTC QLQ-CR29, 29-item European Organisation for Research and Treatment of Cancer Quality of Life Questionaire MDASI, MD Anderson Symptom Inventory; SF-12, short form-12; CARES, Cancer Rehabilitation Evaluation System.

Sexual function was negatively impacted by CRC treatment and while there was some improvement over time many changes are long term and still present 5 years after diagnosis. Raw rates of sexual dysfunction were lower in younger patients when compared with older patients, but the impact that this dysfunction had on younger patients’ QOL and intimate relationships may be more pronounced. These negative effects were seen in both men and women. The negative impact on body image was more pronounced in younger and female patients and this affects both sexual functioning and QOL.

Perl *et al*.^15^ interviewed patients under 40 years of age and found that 40 per cent of patients experienced significant sexual dysfunction. They did find that this improved over time, but never returned to baseline. Using the CARES sexual functioning score they were able to compare scores with breast and prostate cancer patients as well as the normal population. They found that men experience levels of sexual dysfunction just below the mean of those treated with prostate cancer (a cancer that is well known to negatively impact sexual function), sitting in the bottom 41st percentile, whereas, compared with an age-matched general population, the men were in the lowest 17th percentile. EOCRC patients who were women were also found to have significant sexual dysfunction, equivalent to the lowest 13th percentile worse affected when compared with breast cancer patients. The REACCT group^21^ reported much lower rates of sexual dysfunction in EOCRC patients, with 6 per cent of rectal cancer patients reporting sexual dysfunction; they did not, however, state how this was defined.

The qualitative interviews of Blum-Barnett *et al*.^8^ highlighted the strain on sexual function and intimate relationships and difficulty around communication. One patient stated that ‘*for a lot of people under 50,…sexual dysfunction is another thing that’s really hard to talk about. And those are some huge side effects as well that people struggle with*’. That patient wished they had access to more information about sexual dysfunction, a theme that was common in those who were interviewed. Issues with intimate relationships were also common; one participant described the way her cancer diagnosis led to the disintegration of her marriage. Another commented on the difficulties of their husband shifting back and forth between being a caregiver and a romantic lover^8^.

Finally^13^, a cross-sectional survey, investigating the long-term (greater than 5 years) effects of CRC in the USA, reported that sexual dysfunction was a significant problem across all survivors, but rates of sexual dysfunction and impotence were higher in older patients than in younger patients. Poor body image, however, was found to be significantly higher in younger patients compared with older patients. Indeed, it was documented that 24 per cent of those under the age of 40 years experienced a moderate to severe negative impact on their relationship compared with only 11 per cent of those over 40 years (*P* ≤ 0.05)^24^.

### Financial and career impacts

Five studies gave some insight into the impact on patients’ financial well-being and careers^8,15,23–25^ (*Table 4*). The negative effects on financial well-being and career are significant for all people diagnosed with CRC; however, these effects may be more pronounced in EOCRC patients when compared with older patients.

**Table 4:** Impact on financial situation and career

Authors	Age of participants (years)	Key findings relevant to impact on financial situation and career
Sanford *et al.* (2014)^24^	Participants <40 compared with >40	Younger patients reported nearly 3× greater symptom interference with work (OR 2.57 (95% c.i. 1.39,4.74)).
Perl *et al.* (2016)^15^	All participants <40	Patients reported significantly lower scores in ability to perform occupational activities (*P* ≤ 0.05). Financial support and psychosocial support were the types of support most often accessed.
Blum-Barnett *et al.* (2019)^8^	All participants <50	Patients have significant negative effects on career due to various emotional and physical side effects. Prominent themes included impacts on financial situation, costs of treatment, career trajectory, lost earning potential, and reduced performance at work.
de Wind *et al.* (2021)^25^	Participants <45 compared with >45	Patients <45 were at a higher risk of loss of employment 2–4 years after diagnosis compared with older patients (*P* ≤ 0.05). Younger patients also more likely to be on social welfare 0–4 years after diagnosis (*P* ≤ 0.05).
Mo *et al.* (2023)^23^	Patients under <50 compared with 50–64, 65–74, and ≥75	Of patients <50, 90% had scores indicating significant levels of financial toxicity and distress. These figures dropped substantially in the older patient groups (age 50–64, 54%; age 65–74, 40%; and age ≥75, 19% (*P* ≤ 0.001)).

The qualitative study by Blum-Barnett *et al.*^8^ found the negative financial impact to be one of the major themes coming through in their interviews. They divided this into three further categories: effect on career trajectory; lost earning potential; and negative impact on their ability to perform in their work. For many people in their 30s and 40s, this is a pivotal stage in their career. One patient described the effect of their cancer diagnosis on their career as ‘catastrophic’, coming as they were about to make tenure as a university professor. Their study highlighted the often large healthcare bills for some patients, who after surviving their diagnosis then have the stress of paying for their treatment, with one patient stating ‘*Once you realize “I’m going to survive this,” you need to worry about how you’re going to pay for everything.*’ Those patients who were in mentally or physically demanding jobs found they were simply not able to perform in their jobs as they were previously due to the side effects of cancer treatment^8^.

A study^23^ that measured non-metastatic CRC patients’ levels of financial toxicity, using the comprehensive score for financial toxicity (COST)^29^, found that younger age was associated with higher levels of financial toxicity. Of patients under 50 years, 90 per cent scored as having a high level of financial distress; this proportion dropped substantially in the older age groups (age 50–64 years, 54 per cent; age 65–74 years, 40 per cent; and age greater than or equal to 75 years, 19 per cent (*P* ≤ 0.001))^23^.

de Wind *et al*.^25^ looked at the longer-term impacts of CRC on employment by comparing the rates of employment and other financial measures against a control group without CRC. They found that EOCRC patients employed at the time of diagnosis were more likely to be out of paid employment 2–4 years after diagnosis compared with older patients still of working age (HR 1.21, *P* ≤ 0.05). Younger patients were also significantly more likely to be on social welfare up to 4 years after diagnosis (HR 1.31, *P* ≤ 0.05)^25^.

A few studies in this review reported that younger patients were significantly more likely than older patients to have their ability to work impacted by their CRC diagnosis, for example one conducted using the MDASI score^24^ found younger patients were more likely to report symptoms interfering with work compared with older patients, after adjusting for demographics and tumour characteristics, treatments, performance status, and time since diagnosis (adjusted OR 2.57, *P* ≤ 0.01)^24^. Another study^15^ measured the impact on patients’ ability to perform occupational duties using the CARES questionnaire and found that younger patients were significantly more likely to report worse scores measuring occupational activities compared with pretreatment (*P* ≤ 0.05)^15^.

### Emotional distress

The effect on emotional distress was the most widely reported outcome, with eight studies reporting on this issue^8,13,16,17,19,20,24,26^. Studies showed high rates of emotional distress after CRC diagnosis and treatment in patients of all ages. Of the six studies that compared the effect on younger *versus* older patients, four found that the impact was more pronounced in younger patients, with the remaining two finding no significant difference between age groups (*Table 5*).

**Table 5:** Impact on emotional distress

Authors	Age of participants (years)	Scoring tools used	Key findings relevant to impact on emotional distress
Bailey *et al.* (2014)^13^	Participants <50 compared with >50	EORTC QLQ-CR29	Younger patients reported worse rates of anxiety (*P* ≤ 0.05).
Sanford *et al.* (2014)^24^	Participants <40 compared with >40	MDASI	Younger patients reported greater symptom interference with mood (41% in younger patients compared with 15% in older patients) (*P* ≤ 0.01).
Aminisani *et al.* (2017)^16^	Participants <50 compared with >50	HADS, EORTC QLQ-C30	No statistically significant differences in rates of depression, anxiety or global quality of life.
Adams *et al.* (2016)^26^	Participants <50 compared with >50	VR-12	Patients >50 less likely to have mental component summary scores below the 10th percentile (OR 0.37 (95% c.i. 0.15,0.92)). They found that patients with a very low mental component summary score (<10th percentile) had a higher rate (HR 1.98 (95% c.i. 1.19,3.28)).
Blum-Barnett *et al.* (2019)^8^	All participants <50	Qualitative interviews	Early-onset colorectal cancer patients have significant negative effects on quality of life due to various emotional side effects. Prominent themes included anxiety, worry about children, and worry about lack of information.
Aminisani *et al.* (2021)	Participants <50 compared with >50	HADS	No statistically significant difference in rates of depression; however, there were higher rates of anxiety in younger patients (OR 3.12, *P* ≤ 0.05).
Miller *et al.* (2021)^19^	All participants <40	FACT-C	Very low emotional well-being scores using FACT-C (11.67 out of 24). Scores were slightly higher in patients who were 19–36 months past diagnosis compared with patients who were 9–18 months past diagnosis, implying some improvement over time (*P* ≤ 0.05).
Boehmer *et al.* (2021)^20^	Participants <50 compared with >65	EORTC QLQ-CR29	Whilst patients <50 compared with patients >65 had a statistically significant lower physical quality of life (*P* ≤ 0.05), a lower, but not statistically significant, mental quality of life was found (*P* ≥ 0.05).

EORTC QLQ-CR29, 29-item European Organisation for Research and Treatment of Cancer Quality of Life Questionaire; MDASI, MD Anderson Symptom Inventory; HADS, Hospital Anxiety and Depression Scale; EORTC QLQ-C30, 30-item European Organisation for Research and Treatment of Cancer Quality of Life Questionaire; VR-12, Veterans RAND 12; FACT-C, Functional Assessment of Cancer Therapy—Colorectal.

In research performed^19^ using the FACT-C score in patients aged 18–39 years, the lowest scores were in emotional well-being, scoring only 11.7 out of 24. Emotional well-being was higher in patients who were 19–36 months past diagnosis compared with patients who were 9–18 months past diagnosis, implying some improvement over time^19^. In the qualitative study of Blum-Barnett *et al.*^8^, EOCRC patients described many emotional side effects of their cancer diagnosis, which were grouped into themes of emotional stress, strained relationships, and distress due to missing information.

Two studies^13,17^ both found that younger patients had significantly higher rates of anxiety when compared with older patients, although one of these found no difference in the rate of depression^17^. Another study^24^, using a different tool (MDASI), found that 41 per cent of younger patients reported moderate to severe symptom interference with mood compared with just 15 per cent of older patients (*P* ≤ 0.05). They also reported higher general levels of distress (38 per cent *versus* 17 per cent (*P* ≤ 0.01)).

In one report^24^ patients were surveyed over 5 years from diagnosis and it was found that older patients were significantly less likely to be in the lowest decile of the mental component score (OR 0.37 (95 per cent c.i. 0.15 to 0.92)). They also found being in the lowest decile of the mental component score was associated with increased rate (HR 1.98 (95 per cent c.i. 1.19 to 3.28)).

Two studies that compared younger patients with older patients found no statistically significant difference in mental well-being measures; one^20^ showing a worse mental component summary score of the SF-12 QOL tool not reaching significance, and the other^16^ reporting no statistically significant difference in the rate of anxiety or depression measured using the Hospital Anxiety and Depression Scale (HADS)-A and HADS-D scores between EOCRC patients and older patients.

### Social and family impacts

CRC diagnosis and treatment negatively affect patients in terms of social and family functioning. Five studies reported on these aspects^8,14,15,19,24^ (*Table 6*).

**Table 6:** Impact on social and family functioning

Authors	Age of participants (years)	Key findings relevant to impact on social and family functioning
Sanford *et al.* (2014)^24^	Participants <40 compared with >40	Younger patients reported greater interference with their social relationships with others (OR 3, *P* ≤ 0.01).
Mack *et al.* (2016)^14^	Participants <40 compared with >40	Younger patients had more worries about time away from families (*P* = 0.002). Younger patients’ decisions about treatment were more likely to be informed by worries about time away from families.
Perl *et al.* (2016)^15^	All participants <40	Of the patients, 40% reported significantly lower scores in social functioning and caring for children (*P* ≤ 0.05).
Blum-Barnett *et al.* (2019)^8^	All participants <50	Early-onset colorectal cancer patients have significant negative effects on quality of life due to various emotional reasons. Prominent themes included strained personal relationships and worry about children.
Miller *et al.* (2021)^19^	All participants <40	Social well-being scores using FACT-C very low (14.88 out of 28). No difference in social well-being scores when comparing the group 6–18 months after diagnosis with the group 19–36 months after diagnosis.

FACT-C, Functional Assessment of Cancer Therapy—Colorectal.

Blum-Barnett *et al*.^8^ described the negative social impact of the diagnosis, noting the strain it can place on a patient’s ability to perform their role in their family, and Miller *et al*.^19^ reported the mean score in social well-being using the FACT-G QOL tool to be 14.88 out of a possible 28, indicating significantly impacted social well-being. The social well-being scores were not significantly different between the groups interviewed at 6–18 and 19–36 months past diagnosis, suggesting this may be a long-term problem for these patients.

When the focus was on patients all under 40 years, significantly lower scores describing coping with children compared with before treatment were reported^15^.

Furthermore, when comparing EOCRC patients with older patients it was reported that younger patients were significantly more likely to report significant worry about time away from family (*P* = 0.002), and that this was more likely to influence their decision-making around treatments^14^. EOCRC patients were also found to be three times more likely to report symptoms interfering with their relationships with others (OR3.04, *P* ≤ 0.01) compared with older patients^24^.

## Discussion

CRC is generally a disease of older adults, with a median age of about 70 years; however, over recent years, an increasing incidence of EOCRC patients has been described. The pathophysiology, clinical characteristics, and behaviour of EOCRC are different from those of LOCRC^3,30^. This review has found that the psychosocial impacts of CRC are poorly investigated in EOCRC. Overall, the literature is inconclusive; however, there is some evidence that global QOL, alongside psychosocial impacts related to career, finances, relationships, emotional well-being, social well-being, and family well-being, is disproportionately affected in EOCRC patients compared with LOCRC patients. Of the studies included in our review that directly compared the effect of CRC treatment and diagnosis on overall QOL between EOCRC and LOCRC patients, three^18,20,22^ of four found QOL to be more adversely affected in EOCRC patients and the remaining study^16^ found no difference.

The reasons why QOL may be more affected in younger patients are not clear. Younger patients are generally physically fitter and stronger, but this clearly does not translate into an improved psychosocial impact. Factors that were found to increase the risk of low QOL in the included studies were having concurrent chemotherapy or radiation^15^, cancer recurrence, lower socioeconomic status, stage at diagnosis, time from diagnosis, being female, and having more co-morbidities^14,15,19,31^. None of these factors explains the age disparities. When comparing the global FACT-C scores of patients from one of the studies included in the present review (patients aged less than 40 years)^19^ with a comparable study in older patients (median age of 66.4 years) there is a stark difference, with the younger patients scoring a mean of 67.7 out of a possible 136, compared with the older patients averaging 111.9 out of 136^32^.

One possible explanation could be ‘the gap’ hypothesis^33,34^, which describes negative QOL often being the result of the ‘gap’ between patients' hopes and aspirations and their lived reality. Younger patients are surrounded by healthy peers with fewer problems in life and therefore have very high expectations for what they can achieve (and what they should be achieving). Older patients, however, have more reserved expectations and ideas about where their life should be, having been exposed to more illness in themselves and their peers, and therefore the ‘gap’ between their expectations and their reality is less than in younger patients. Another possibility is related to the theory of response shift^35^, a phenomenon where patients have to recalibrate and reprioritize their values in response to a major life insult, leading to a subjective increase in reported QOL as a result; this effect may be more pronounced in older patients^34^.

General well-being is also likely to naturally fluctuate as people age. One large study involving over 350 000 participants has shown that overall well-being follows a U curve pattern, naturally increasing after 50–85 years of age. They also found that some negative emotions such as stress, anger, and worry all decrease as we age^36^.

Sexual dysfunction is a known side effect of CRC and its treatment, especially rectal cancers, which are increasingly prevalent in EOCRC^37^. The rates of sexual dysfunction negatively impact on patient QOL^38^. This is particularly relevant to younger patients, with a study of patients with CRC showing that, compared with older patients, maintaining a sex life was significantly more important^7^. One study^15^ showed that men with EOCRC under 40 years had rates of erectile dysfunction far worse than members of the general population of the same age. Whilst raw numbers of sexual dysfunction are often higher in older patients^4^, care must be taken interpreting these figures as rates of erectile dysfunction naturally increase with age^39^. What is more important is not the raw rates of sexual dysfunction but rather the impact that sexual dysfunction is having on those patients. For example, a study of CRC survivors published in 2022 found that older patients were less interested in sex compared with younger patients and complained of less overall sexual dysfunction^40^. Another reported that despite the rates of sexual dysfunction in older men being higher, younger men were more likely to be distressed by sexual disinterest^41^. Whilst the sexual complications in women are perhaps less well publicized than in men, young women are particularly vulnerable to this, with a study of female patients with rectal cancer recently showing that 86 per cent of women reported body image issues, with 40 per cent saying one of these issues was severe, and 51 per cent complaining of pain during vaginal penetration^42^. While avoiding a stoma is still important even to the very old (greater than 80 years)^43^, the negative impact of having an ostomy has been shown to be more pronounced in younger and female patients^10^. Ostomies have been shown to adversely affect both sexual function and body image in CRC patients^44^.

The mechanisms of sexual dysfunction in CRC survivors are complex. There are both the physical and medical complications of treatment, as well as the immense psychological stress that comes with a cancer diagnosis and treatment. These all can lead to loss of the ability to have and desire sex, as well as causing anxiety and depression^45^.

Financial and career stresses are more common in younger cancer patients compared with older patients. Up to 90 per cent of EOCRC patients are found to report significant financial toxicity^23^. Some studies surveying multiple QOL domains found that the largest differences between young and old patients were found in stress around financial difficulties and social functioning^46^. This is particularly relevant as a study of 2000 adult cancer survivors reported that financial distress was the strongest predictor of lower QOL^47^. They are also more likely to be out of paid work compared with older patients (still of working age) 2–4 years after their diagnosis^25^.

Being in the most productive years of life and having to put everything on hold to go through gruelling cancer treatment can lead to significant financial distress and loss of earnings. One of the studies reported herein^8^ summarized the different aspects of financial impact as not only loss of earnings for that interval but also described what can be a significant long-term cost of reduced career progression and subsequent long-term higher income. Another factor was that many people in high-functioning jobs stated that they did not feel they could go back to the same level of functioning at work after treatment. These factors compound the significant costs of treatment in countries without funded healthcare like the USA. Perl *et al*.^15^ showed that financial services were the most accessed support service for younger patients, again highlighting the importance of these services for EOCRC patients. The levels of financial impact may be worse in countries where healthcare is not free, and indeed most of the papers reviewed on this aspect were from the USA^8,13,15,24^. However, there are still several papers we found from countries with free healthcare such as Australia^9^, Germany^48^, and the Netherlands^25^, showing that these financial impacts are due to more than just the costs of treatment, and likely as much related to lost earnings and negative impact on career.

Levels of emotional distress, particularly anxiety, may also disproportionately affect younger patients. Higher rates of anxiety and depression are related to worse QOL^16^. Causes for this are likely multifactorial. Earlier in adulthood is a more complex time with regards to identity development and self-confidence. The ability to perform or not perform tasks can threaten a person’s sense of self-worth. Some of this could also be explained by higher rates of fear of cancer recurrence, which has been reported in other studies to be higher in younger patients^49^, and fear of cancer recurrence has been shown to be associated with lower QOL. Higher levels of family-related concerns in younger patients were also a common finding. The distress about time away from families was reported to be associated with having dependent children at home, which is far more common in younger patients^14^. The association here is easily explained, but it will be one of the major concerns for many patients with young children as they navigate both coming to terms with a difficult diagnosis and having to explain the significance of the situation to dependent children. These problems may also continue for a long time after treatment, with one study in the present review^19^ reporting no improvement in social well-being scores between the groups interviewed 6–18 and 19–36 months after diagnosis.

Another factor may be that physical symptoms are felt more by younger patients, and this spills over into worse QOL and more emotional distress. The perception of pain changes as the body ages^50^, and a recent systematic review of changes in pain perception related to age found that pain threshold increases with age, but pain tolerance does not, confirming that younger people feel more pain at lower pain intensities^50^. Indeed, young patients with CRC were found more likely to report several physical symptoms after treatment and were found more likely to report moderate to severe pain interfering with their ability to work^24^.

The literature is mixed regarding interventions for improving QOL. A systematic review published in 2017^51^ reported that 8 of the 14 RCTs they reviewed produced little to no effects on outcomes. Possible effective interventions they found included written and verbal emotional expression, progressive muscle relaxation training, and a self-efficacy enhancing intervention. Other interventions include mindfulness^52^, exercise programmes^53^, and activity trackers^54^.

Evidence regarding interventions that will improve QOL in EOCRC patients is so far lacking and is an area that needs further research. Nevertheless, there is some research showing that younger patients are more likely to utilize support than older patients, and therefore they may be more likely to uptake any support or any effective intervention that does arise^55^. There are different barriers to interventions regarding age, with older patients finding interventions harder due to physical ability, and younger patients finding interventions harder due to lack of time^56^.

This review has limitations. Eleven of the studies included were cross-sectional, with such studies having limitations. They are only able to show associations between factors, they do not show temporal association, they are prone to participation or response bias, and it is difficult to make a causal inference. One of these studies conducted a survey through social media, which introduces further possible selection bias^19^.

Most studies included used validated scoring systems to measure things like QOL, depression, anxiety, sexual function, and symptoms. However, validated scoring systems all have their own strengths and weaknesses^28^.

There was significant heterogeneity across the studies. While all included patients under 50 years, some were comparing patients under 40 years and some papers included a proportion of patients with cancers other than CRC such as lung^14^ and stomach^18^ cancer. Papers reporting on QOL all used different scoring tools, or a qualitative interview. Lastly, there was a wide range of time points after diagnosis assessed. All these factors mean that any conclusions drawn from across these results need to be interpreted with caution.

Aside from one study^25^, none had normal population controls. Using some validated scores, we have some ability to reference values from other studies, which can be a helpful comparison, but is inferior to direct comparisons of control groups within the same populations.

The strength of the review was that it included a wide search strategy, including both studies involving EOCRC patients only, but also studies of all age groups provided they reported their results analysed by age. The included studies were of a high quality when assessed using the JBI critical appraisal tools.

Clinicians, when seeing EOCRC patients, should routinely enquire about patient mental health, well-being, sexual issues, and financial issues, and refer for support when required. While some studies reported that certain symptoms do improve with time, studies including only long-term cancer survivors still found significant deficits, which highlights that many issues will be with patients long term^13^. Support specific to this age group that addresses concerns as described above should be considered to improve the QOL in patients with EOCRC.

## Supplementary Material

zrad030_Supplementary_Data

## Data Availability

All data supporting the findings of this systematic review are from previously reported studies, which have been cited.
