# Supplementary material for: Quality of life in early-onset colorectal cancer patients: systematic review
Source: BJS Open. 2023 May 8;7(3):zrad030. doi: 10.1093/bjsopen/zrad030 (PMC10165061; doi:10.1093/bjsopen/zrad030)
Supplement: zrad030_Supplementary_Data [file zrad030_supplementary_data.docx]

**Quality of life in early onset colorectal cancer patients: systematic review.**

Authors

Oliver Waddell^1^, MBChB

Jared Mclauchlan^1^, MBChB

Andrew McCombie^1^, PhD

Tamara Glyn^1^, FRACS

Frank Frizelle^1^, MMedSc, FRACS

Corresponding author

Dr Oliver Waddell

Email: [revilox@gmail.com](mailto:revilox@gmail.com)

Address: Department of Surgery, University of Otago Christchurch, 36 Cashel St, Christchurch Central,

Christchurch, 8011

ORCID ID: 0000-0002-4175-7603

Affiliation

^1^ Department of Surgery, University of Otago, Christchurch.

**Supplementary Materials - Index**

| **Supplementary Methods** |  |
| --- | --- |
| Detailed database search strategies**.** | *pag. 2* |
| **Supplementary Figures and Tables** |  |
| Table S1. Critical appraisal of cross-sectional studies part one | *pag. 3* |
| Table S2. Critical appraisal of Cross-sectional studies part two. | *pag. 4* |
| Table S3. Critical appraisal of Qualitative studies | *pag. 5* |
| Table S4. Critical appraisal of Cohort studies. | *pag. 6* |
| Table S5. Critical appraisal of case-control studies. | *pag. 7* |

**Supplementary Methods**

**Detailed database search strategies.**

The following search strategies were used. Search was undertaken with guidance from experienced librarian.

**Scopus.**

## KEY ( ( ( colorectal  AND  cancer* )  OR  ( bowel  AND  cancer* )  OR  ( colorectal  AND  neoplasm* )  OR  ( bowel  AND  neoplasm* ) )  AND  "quality of life"  AND  survivor )  AND  ( LIMIT-TO ( PUBYEAR ,  2022 )  OR  LIMIT-TO ( PUBYEAR ,  2021 )  OR  LIMIT-TO ( PUBYEAR ,  2020 )  OR  LIMIT-TO ( PUBYEAR ,  2019 )  OR  LIMIT-TO ( PUBYEAR ,  2018 )  OR  LIMIT-TO ( PUBYEAR ,  2017 )  OR  LIMIT-TO ( PUBYEAR ,  2016 )  OR  LIMIT-TO ( PUBYEAR ,  2015 )  OR  LIMIT-TO ( PUBYEAR ,  2014 )  OR  LIMIT-TO ( PUBYEAR ,  2013 )  OR  LIMIT-TO ( PUBYEAR ,  2012 ) )  AND  ( LIMIT-TO ( DOCTYPE ,  "ar" ) )  AND  ( LIMIT-TO ( LANGUAGE ,  "English" ) )

## Filtered studies to years 2012-2022 in the English language.

## Pubmed.

## Search: ( ( ( colorectal  AND  cancer* )  OR  ( bowel  AND  cancer* )  OR  ( colorectal  AND  neoplasm* )  OR  ( bowel  AND  neoplasm* ) )  AND  "quality of life"  AND  survivor ) AND (y_10[filter]) AND (English[filter])

## Filtering for studies in the past 10 years and in English.

## Ovid.

## Searching Medline, Embase and Cochrane central register of controlled trials.

## #1. Bowel cancer*.mp

## #2. Bowel Neoplasm*.mp

## #3. Colorectal cancer*.mp

## #4. Colorectal neoplasm*.mp

## #5. #1 or #2 or #3 or #4

## #6. “Quality of life”

## #7. Survivor

## #8. #5 and #6 and #7

## CINAHL.

## S1. TX Bowel cancer*

## S2. TX Bowel Neoplasm*

## S3. Colorectal cancer*

## S4. Colorectal neoplasm*

## S5. S1 OR S2 OR S3 OR S4

## S6. “Quality of life”

## S7. Survivor

## S8. S5 and S6 and S7

## Excluding Medline records. Limit to English language, peer reviewed articles published 2012-2022.

**Supplementary Figures and Tables**

**Table S1. Critical appraisal of cross-sectional studies part one**

| Questions | Bailey (2014) | Perl  (2016) | Mack (2016) | Aminisani (2017) | Kobayashi  (2020) | Aminisani (2021) |
| --- | --- | --- | --- | --- | --- | --- |
| 1.       Were the criteria for inclusion in the sample clearly defined? | yes | yes | yes | yes | yes | yes |
| 2.       Were the study subjects and the setting described in detail? | yes | yes | yes | yes | yes | yes |
| 3.       Was the exposure measured in a valid and reliable way? | yes | yes | yes | yes | yes | yes |
| 4.       Were objective, standard criteria used for measurement of the condition? | yes | yes | yes | yes | yes | yes |
| 5.       Were confounding factors identified? | yes | yes | yes | yes | yes | yes |
| 6.       Were strategies to deal with confounding factors stated? | yes | yes | yes | yes | yes | yes |
| 7.       Were the outcomes measured in a valid and reliable way? | yes | yes | Unclear | yes | yes | yes |
| 8.       Was appropriate statistical analysis used? | yes | yes | yes | yes | yes | yes |
| % of criteria met | 100% | 100% | 87.5% | 100% | 100% | 100% |
| Comments |  | 20% of cancers were GI tract other than CRC | Did not use validated scoring systems instead used their own questionnaire. |  | Included lung and stomach cancer, however 47.5% of total patients were CRC. |  |
| Include | yes | yes | yes | Yes | yes | yes |

**Table S2. Critical appraisal of Cross-sectional studies part two**.

| Questions | Miller  (2021) | Boehmer (2021) | REACCT collaborative,  (2022) | Potosky, (2022) | Mo, (2023) |
| --- | --- | --- | --- | --- | --- |
| 1.       Were the criteria for inclusion in the sample clearly defined? | yes | yes | yes | Yes | Yes |
| 2.       Were the study subjects and the setting described in detail? | yes | yes | yes | Yes | yes |
| 3.       Was the exposure measured in a valid and reliable way? | yes | yes | yes | Yes | yes |
| 4.       Were objective, standard criteria used for measurement of the condition? | yes | yes | yes | Yes | yes |
| 5.       Were confounding factors identified? | yes | yes | yes | yes | yes |
| 6.       Were strategies to deal with confounding factors stated? | yes | yes | yes | yes | yes |
| 7.       Were the outcomes measured in a valid and reliable way? | yes | yes | unclear | yes | yes |
| 8.       Was appropriate statistical analysis used? | yes | yes | yes | yes | yes |
| % of criteria met | 100% | 100% | 87.5% | 100% | 100% |
| Comments | Social media survey, but authors took several steps to minimise risk of fraudulent answers. |  | Did not state how sexual dysfunction was measured |  |  |
| Include | yes | yes | yes | yes |  |

**Table S3. Critical appraisal of Qualitative studies**

| Questions | Blum-Barnett (2019) |
| --- | --- |
| 1.         Is there congruity between the stated philosophical perspective and the research methodology? | yes |
| 2.         Is there congruity between the research methodology and the research question or objectives? | yes |
| 3.         Is there congruity between the research methodology and the methods used to collect data? | yes |
| 4.         Is there congruity between the research methodology and the representation and analysis of data? | yes |
| 5.         Is there congruity between the research methodology and the interpretation of results? | yes |
| 6.         Is there a statement locating the researcher culturally or theoretically? | no |
| 7.         Is the influence of the researcher on the research, and vice- versa, addressed? | unclear |
| 8.         Are participants, and their voices, adequately represented? | yes |
| 9.         Is the research ethical according to current criteria or, for recent studies, and is there evidence of ethical approval by an appropriate body? | yes |
| 10.     Do the conclusions drawn in the research report flow from the analysis, or interpretation, of the data? | yes |
| % of criteria met | 80% |
| Comments | Small number of participants (n=14). All participants had health insurance possibly skewing the findings about financial difficulties. |
| Include | Yes - despite some limitation still provides good qualitative insights into a group of EOCRC patients. Need to be cautious generalising results across all EOCRC. |

**Table S4. Critical appraisal of Cohort studies.**

| Questions | Adams (2016) |
| --- | --- |
| 1.       Were the two groups similar and recruited from the same population? | yes |
| 2.       Were the exposures measured similarly to assign people to both exposed and unexposed groups? | Unclear - Exposure was differing HRQOL scores. The participants were then analysed in groups based on their HRQOL scores. |
| 3.       Was the exposure measured in a valid and reliable way? | yes |
| 4.       Were confounding factors identified? | yes |
| 5.       Were strategies to deal with confounding factors stated? | yes |
| 6.       Were the groups/participants free of the outcome at the start of the study (or at the moment of exposure)? | yes |
| 7.       Were the outcomes measured in a valid and reliable way? | yes |
| 8.       Was the follow up time reported and sufficient to be long enough for outcomes to occur? | yes |
| 9.       Was follow up complete, and if not, were the reasons to loss to follow up described and explored? | yes |
| 10.   Were strategies to address incomplete follow up utilized? | yes |
| 11.   Was appropriate statistical analysis used? | yes |
| % of criteria met | 91% |
| Comments | Prospective study following a single Cohort of CRC patients, measuring HRQOL and then following to mortality over time. Essentially a mixed methods study. The data on HRQOL scores was cross-sectional, the analysis of the effect of HRQOL on mortality was cohort. |
| Include | Yes |

**Table S5. Critical appraisal of case-control studies.**

| Questions | Sanford (2013) | De Wind (2021) |
| --- | --- | --- |
| 1.       Were the groups comparable other than the presence of disease in cases or the absence of disease in controls? | yes | yes |
| 2.       Were cases and controls matched appropriately? | yes | yes |
| 3.       Were the same criteria used for identification of cases and controls? | yes | yes |
| 4.       Was exposure measured in a standard, valid and reliable way? | yes | yes |
| 5.       Was exposure measured in the same way for cases and controls? | yes | yes |
| 6.       Were confounding factors identified? | yes | yes |
| 7.       Were strategies to deal with confounding factors stated? | yes | yes |
| 8.       Were outcomes assessed in a standard, valid and reliable way for cases and controls? | yes | yes |
| 9.       Was the exposure period of interest long enough to be meaningful? | no - only 4-5 weeks elapsed between | yes |
| 10.   Was appropriate statistical analysis used? | yes | yes |
| % of criteria met | 90% | 100% |
| Comments | Very short time frame between first and second interview. Study still included however as it provides good comparisons in symptoms and QOL in patients under 40 compared with over 40 at each time point regardless of little change between the two time points. |  |
| Include | yes | yes |
